# Midkine Is Elevated After Multiple Trauma and Acts Directly on Human Cardiomyocytes by Altering Their Functionality and Metabolism

**DOI:** 10.3389/fimmu.2019.01920

**Published:** 2019-08-21

**Authors:** Ina Lackner, Birte Weber, Meike Baur, Melanie Haffner-Luntzer, Tim Eiseler, Giorgio Fois, Florian Gebhard, Borna Relja, Ingo Marzi, Roman Pfeifer, Sascha Halvachizadeh, Miriam Lipiski, Nikola Cesarovic, Hans-Christoph Pape, Miriam Kalbitz, TREAT Research Group

**Affiliations:** ^1^Department of Traumatology, Hand, Plastic, and Reconstructive Surgery, Center of Surgery, University of Ulm, Ulm, Germany; ^2^Institute of Orthopedic Research and Biomechanics, University of Ulm, Ulm, Germany; ^3^Department of Internal Medicine I, University of Ulm, Ulm, Germany; ^4^Institute of General Physiology, University of Ulm, Ulm, Germany; ^5^Department of Trauma, Hand and Reconstructive Surgery, Goethe University Frankfurt, Frankfurt, Germany; ^6^Department of Trauma, University Hospital of Zurich, Zurich, Switzerland; ^7^Department of Surgical Research, University Hospital of Zurich, Zurich, Switzerland

**Keywords:** polytrauma, cardiac dysfunction, fracture treatment, damage associated molecular pattern, toll-like receptor, toll-like receptor signaling, prevention cardiac injury, CytoSorb® 300

## Abstract

**Background and Purpose:** Post-traumatic cardiac dysfunction often occurs in multiply injured patients (ISS ≥ 16). Next to direct cardiac injury, post-traumatic cardiac dysfunction is mostly induced by the release of inflammatory biomarkers. One of these is the heparin-binding factor Midkine, which is elevated in humans after fracture, burn injury and traumatic spinal cord injury. Midkine is associated with cardiac pathologies but the exact role of Midkine in the development of those diseases is ambiguous. The systemic profile of Midkine after multiple trauma, its effects on cardiomyocytes and the association with post-traumatic cardiac dysfunction, remain unknown.

**Experimental Approach:** Midkine levels were investigated in blood plasma of multiply injured humans and pigs. Furthermore, human cardiomyocytes (iPS) were cultured in presence/absence of Midkine and analyzed regarding viability, apoptosis, calcium handling, metabolic alterations, and oxidative stress. Finally, the Midkine filtration capacity of the therapeutic blood absorption column CytoSorb ®300 was tested with recombinant Midkine or plasma from multiply injured patients.

**Key Results:** Midkine levels were significantly increased in blood plasma of multiply injured humans and pigs. Midkine acts on human cardiomyocytes, altering their mitochondrial respiration and calcium handling *in vitro*. CytoSorb®300 filtration reduced Midkine concentration *ex vivo* and *in vitro* depending on the dosage.

**Conclusion and Implications:** Midkine is elevated in human and porcine plasma after multiple trauma, affecting the functionality and metabolism of human cardiomyocytes *in vitro*. Further examinations are required to determine whether the application of CytoSorb®300 filtration in patients after multiple trauma is a promising therapeutic approach to prevent post-traumatic cardiac disfunction.

## Introduction

According to the World Health Organization (WHO), trauma accounts for 10% of deaths and 16% of disabilities worldwide (1). Multiple trauma in humans (Injury Severity Score, ISS ≥ 16) are characterized by a massive release of different inflammatory biomarkers, such as cytokines, and damage associated molecular patterns (DAMPs). This damage affects different organs of the body and can trigger whole-body inflammation after trauma (2, 3). A substantial release of these trauma-dependent molecules is associated with the development of the so-called systemic inflammatory response syndrome (SIRS) and the multiple organ dysfunction syndrome (MODS), which are both associated with an increased mortality (4, 5). Many of the released inflammatory cytokines and DAMPs were recently shown to be cardio-depressive by acting on cardiomyocytes (CMs), altering their calcium handling, redox balance, signaling transduction, and finally resulting in post-traumatic cardiac dysfunction (6, 7). One inflammatory cytokine is the heparin-binding growth- and differentiation factor Midkine (Mdk). Increased Mdk expression is associated with different traumatic conditions such as bone fracture, burn injury, traumatic spinal cord injury, and sepsis (8–11). Increased Mdk in human blood can persist for overall 42 days after fracture (11). Furthermore, Mdk impairs fracture healing by reducing bone formation and increasing neutrophil infiltration during the fracture healing process (12, 13). However, the trauma-dependent elevation of Mdk in multiply injured patients as well as the exact impact of Mdk on the heart after trauma remains unclear. In patients with chronic heart failure, circulating Mdk increases significantly and is regarded as a novel marker, predicting different cardiac events (14, 15). Moreover, Mdk plays a role in ischemic heart injury, myocardial infarction and cardiac hypertrophy (16–18). Nevertheless, the function of Mdk in these different pathologies is still controversial, because in some cases such as ischemic heart injury, chronic heart failure and myocardial infarct, Mdk has positive effects by improving cell survival and cardiac function, inducing angiogenesis and reducing detrimental remodeling (17, 19, 20). In contrast, Mdk reduces cellular survival and induces pathological remodeling as well as fibrosis in patients with cardiac hypertrophy (18). Consequently, the exact effect of Mdk on the heart is ambiguous since Mdk can have beneficial and detrimental effects in cardiac pathology. The function of Mdk as an inflammatory cytokine on the heart during trauma especially requires clarification. After all, Mdk might be a potential therapeutic option in cardiac diseases as well as in the treatment and prevention of post-traumatic cardiac injury (21, 22). Mdk has been shown to play an important role in active myocarditis in patients and in experimental autoimmune myocarditis in mice (23). In these instances, Mdk promotes the recruitment of polymorphonuclear neutrophils (PMNs) and the production of neutrophil extracellular traps (NETs) in cardiac tissues, resulting in impaired systolic function (23). Increased activation and recruitment of neutrophils in cardiac tissue were also observed in humans after trauma and in experimental blunt chest trauma models in rats. In addition, it is linked to increased systemic levels of extracellular histones by NETosis, leading to cardiac dysfunction (24, 25).

In this study, we investigate the Mdk elevation in blood circulation after multiple trauma in pigs and humans. We further aim to thoroughly examine the effects of Mdk on human CMs. With regards to therapeutic options for posttraumatic cardiac dysfunction, the study aims to investigate the usage of CytoSorb® 300 hemadsorption. In clinical settings, CytoSorb® 300 hemadsorption improved the outcome of patients with endotoxemia, necrotizing fasciitis, septic shock, and cardiac surgery (26–29). Furthermore, CytoSorb® hemadsorption resulted in immediate hemodynamic stabilization and increased survival rates in patients with multiple organ failure (30). CytoSorb® 300 consists of highly porous (styrene-co-divinylbenzene) hemadsorbent polymer beads, which can remove substances within 10–60 kDa of molecular weight, such as complement factor 5a, cytokines DAMPs and pathogen associated molecular patterns (PAMPs), from circulating blood (26, 31). Similarly, the high-mobility group box 1 protein (HMGB1) can be removed from blood in a time dependent manner (31). Lastly, the study examines the capacity of CytoSorb® 300 to filtrate Mdk, which may be used as a therapeutic approach for preventing and handling post-traumatic cardiac dysfunction.

## Materials and Methods

### Human Blood Samples

Human plasma from 11 multiply injured patients with a history of acute blunt or penetrating trauma and an ISS ≥ 16 was collected after hospital admission in the University Hospital of the Goethe-University Frankfurt with institutional ethics committee approval (312/10), in accordance with the Declaration of Helsinki and following the Strengthening the Reporting of Observational studies in Epidemiology (STROBE)-guidelines (32). All enrolled patients either signed the written informed consent form or written informed consent was obtained from the nominated legally authorized representative of the participants in accordance with ethical standards. Exclusion criteria were the patients being younger than 18 or older than 80 years, presenting severe burn injury, acute myocardial stroke, cancer or chemotherapy, immunosuppressive drug therapy, HIV, infectious Hepatitis, acute CMV infection, and/or thromboembolic events. Control blood samples were collected from healthy volunteers (*n* = 6, 50:50 female male, no comorbidities). Randomization of the groups was not possible during the sample collection. Blood samples were withdrawn in ethylenediaminetetraacetic acid (EDTA) tubes (Sarstedt, Nürmbrecht, Germany) directly after admission. The samples were kept on ice until centrifugation at 2,100 g for 15 min. Then, the supernatant was collected and stored at −80°C until assay.

### Animals

This study presents partial results obtained from a large animal porcine multiple trauma model, conducted by the TREAT research group.

The animal housing and experimental protocols were approved by the Cantonal Veterinary Department, Zurich, Switzerland, under license no. ZH 138/2017, and were in accordance with Swiss Animal Protection Law. Housing and experimental procedures also conformed to the European Directive 2010/63/EU of the European Parliament and of the Council on the Protection of vertebrate animals used for scientific purposes (Council of Europe no. 123, Strasbourg 1985) and to the Guide for the Care and Use of Laboratory Animals (Institute of Laboratory Animal Resources, National Research Council, National Academy of Sciences, 2011). Twenty-five male pigs weighting 50 ± 5 kg (*Sus scrofa domestica*) were included in the study (mean height, snout-tail length: 123,6 cm). Animals were held in a controlled environment with 21 ± 3°C room temperature (50% humidity), with a light/dark cycle of 12 h. Water was available for animals *ad libitum*. General instrumentation, anesthesia and trauma induction were described previously by Horst et al. (33).

### Analgesia and Anesthesia

For premedication, pigs received an intramuscular injection with ketamine (20 mg/kg body weight), azaperone (1–2 mg/kg body weight) and atropine (0.1–0.2 mg/kg body weight). Anesthesia was performed by intravenous application of propofol (2,6-diisopropylphenol) (1–2 mg/kg body weight). Anesthesia was maintained during the study period with propofol (5–10 mg/kg/h). Pain medication was ensured by sufentanyl (1 μg/kg/h) perfusion over the whole observation period.

### Multiple Trauma in Pigs

Analgesia and Anesthesia of the animals was maintained during the whole procedure.

Pigs underwent either multiple trauma (*n* = 20) or sham-procedure (*n* = 5). Multiple trauma includes a combination of a penetrating thorax trauma, laparotomy, liver laceration, femur fracture, and hemorrhagic shock (ISS ≥ 27). Control animals underwent sham-procedure (*n* = 5). Femur fracture was induced by a bolt gun (Blitz-Kernen, turbocut JOBB GmbH, Germany), positioned on the mid third of the left femur. The gun was loaded with cattle-killing cartridges (9 x 17; DynamitNobel AG, Troisdorf, Germany). For introduction of blunt chest trauma, a pair of panels (steel 0.8 cm, lead 1.0 cm thickness) was placed on the right dorsal lower chest. A shock wave was induced by a bolt shot (Blitz-Kerner, turbocut JOBB GmbH, Germany), which was applied onto the panel using cattle-killing cartridges as previously described (34, 35). Midline-laparotomy was performed by exploring the right upper liver lobe. Penetrating hepatic injury was induced by cross-like incision halfway through the liver tissue. After a short period of uncontrolled bleeding (30 s), liver package was performed. Directly after the hepatic package, pressure-controlled and volume-limited hemorrhagic shock was induced by withdrawing blood until a mean arterial pressure (MAP) of 30 ± 5 mm Hg was reached. Maximal withdrawal amounts to 45% of total blood volume. The reached MAP was maintained for 60 min. At the end of the shock period, animals were resuscitated according to established trauma guidelines (ATLS®, AWMF-S3 guideline on Treatment of Patients with Severe and Multiple Injuries®) by adjusting FiO_2_ and an initial substitution of the withdrawn blood volume with Ringerfundin, fluid maintenance was performed by continuous infusing additional fluids (Ringerfundin, 2 ml/kg body weight/h). Moreover, pigs were rewarmed until normothermia (38.7–39.8°C) was reached. Sham procedure (*n* = 5) included instrumentation and anesthesia but without trauma or hemorrhage. The multiple trauma group (*n* = 20) was randomized in four therapy arms: pigs received either femoral nailing without reaming (*n* = 5), standard reaming (*n* = 5), reamed irrigation and aspiration (RIA I) (*n* = 5) or reamed irrigation and aspiration with reduced diameter and improved control of irrigation and suction (RIA II) (*n* = 5). In all groups a shortened conventional tibia nail was introduced.

### Follow-Up and Euthanasia

Hemodynamic parameters were continuously monitored for 6 h. Pigs were euthanized under deep general anesthesia with intravenous Na-Pentobarbital.

This animal model represents a clinically relevant porcine model of severe multiple trauma (pulmonary contusion, extremity injury, liver laceration) with post-traumatic observation period under ICU conditions (33).

### Sample Collection

Serum and plasma samples were collected at baseline, 4 and 6 h after multiple trauma and kept on ice. After centrifugation (1,500 g for 12 min at 4°C), serum and EDTA-plasma were removed and stored at −80°C until analysis. Heart tissue samples were obtained 6 h after resuscitation. Tissue of the superficial and the luminal left ventricle was fixed with 4% formalin, followed by embedding in paraffin. Furthermore, tissue was quick-frozen in liquid nitrogen, followed by storage at −80°C until analysis.

### Midkine ELISA

For determination of Midkine in human and porcine plasma, as well as for the CytoSorb® 300 experiments, the human Midkine ELISA (R&D Systems, McKinley, MN, USA) was used. All procedures were performed according to manufacturers' instructions. Midkine ELISA was performed by a blinded investigator. Human plasma samples were diluted 1:4 and porcine plasma samples were diluted 1:2.

### ips-Cardiomyocyte Cell Culture

Human cardiomyocytes (iPS) (Cellular Dynamics, Madison, WI, USA) were cultured for 10 days in maintenance medium at 37°C and in an atmosphere of 7% CO_2_, according to manufacturers' recommendations.

### Binding Analysis of FITC-Labeled Midkine

Fluorescein isothiocyanate (FITC) (Sigma Aldrich, St. Louis, MO, USA) was dissolved in DMSO. Two mg/ml Midkine (Dianova, Hamburg, Germany), dissolved in 0.1 M NaHCO_3_ were added to 3 mg/ml fluorescein isothiocyanate (FITC) (Sigma Aldrich, St. Louis, MO, USA) solution and were incubated for 1 h at RT while continuously shaking. Unbound FITC was removed by using SnakeSkin® dialysis tube (ThermoScientific, Waltham, MA, USA). For dialysis, 1X phosphate buffered saline was used. Human CMs were seeded at a density of 6.3 × 10^4^ cells/cm^2^ on ibidi 12-well chamber slides (ibidi, Germany). Afterwards, cells were incubated for 30 and 60 min with 100 ng/ml FITC-labeled Midkine. Cells were washed, fixed with 4% formalin and cell nuclei were counterstained using Hoechst (Sigma Aldrich, St. Louis, MO, USA). Cells were mounted with ProLong® Gold Antifade Mountant (ThermoScientific, Waltham, MA, USA). Cells were analyzed by blinded investigator by using Axio Imager M.2 microscope (Zeiss, Jena, Germany) and the Zeiss ZEN 2.3 software (Zeiss, Jena, Germany). Images were performed with 40x magnification (N.A. 0.75).

### Cell Viability Assay

Cell viability was analyzed using Cell Titer-Glo® Luminescent Cell Viability Assay (Promega, Madison, WI, USA). Cells were seeded with a density of 6.3 × 10^4^ cells/cm^2^ on a 96-well plate and treated with different Midkine concentrations (0.05, 0.1, 1 μg/ml) for 3 h, or with 1 μg/ml for different incubation times (0.5, 1, or 3 h). All procedures were performed according to manufacturers' instructions. For all experiments *n* = 6.

### Troponin I ELISA

Human CMs were seeded with a density of 6.3 × 10^4^ cells/cm^2^ on a 24-well plate and treated for 6 h with 100 ng/ml Midkine at 37°C and 7% CO_2_. Supernatant was collected and troponin I in supernatant was determined by using Human Cardiac Troponin I ELISA (Abcam, Cambridge, UK). All procedures were performed according to manufacturers' instructions. For all experiments *n* = 6.

### Caspase-3/7 Assay

Human cardiomyocytes were seeded with a density of 6.3 × 10^4^ cells/cm^2^ on a 96-well plate and treated with 100 ng/ml Midkine for 6 h at 37°C. Caspase-3/7 activity in human cardiomyocytes was examined by using Caspase-Glo® 3/7 Assay (Promega, Madison, WI, USA). All procedures were performed according to manufacturers' instructions. For all experiments *n* = 6.

### Live Cell Imaging

Live cell imaging was performed using Leica Microscope SP8 and LAS X software (Leica, Wetzlar, Germany). Cells were seeded with a density of 6.3 × 10^4^ cells/cm^2^ on a 96-well plate and were pre-loaded with 5 μM calcium indicator Fluo-3AM (Life Technologies, Carlsbad, CA, USA) and were incubated for 30 min at 37°C and 7% CO_2_. After incubation with Fluo-3AM, cells were analyzed immediately. For measurements, cells were placed in special live cell imaging chamber, adjusted at 37°C and 7% CO_2_. Cells were incubated with 100 ng/ml Mdk for 30 min and calcium signals were recorded and evaluated by using LAS X software. Cell culture medium was used during measurements. Live cell imaging was performed with 63x magnification (N.A. 1.2, water). Calcium peaks were determined and compared to baseline values. For all experiments *n* = 6.

### Calcium Measurements

For calcium measurements, human cardiomyocytes (iPS) were seeded with a density of 6.3 × 10^4^ cells/cm^2^ on ibidi 8-well chambers (ibidi, Germany). Before the measurements, cells were incubated with 100 ng/ml Midkine 60 min before the start of the experiments, as well as for the duration of the experiment. For measurement of changes in intracellular Ca^2+^ concentration, cells were loaded with 5 μM Fura-2 (ThermoScientific, Waltham, MA, USA) for 30 min (in presence of pharmacological compounds if needed). After incubation, cells were washed twice with bath solution (in mM: 140 NaCl; 5.4 KCl; MgCl_2_; 1.8 CaCl_2_; 5.5 Glucose; 5 Hepes; pH = 7.4). Fluorescence imaging was performed on a Cell Observer inverse microscope (Zeiss, Jena, Germany). Cells were illuminated for 90 min at a rate of 2 Hz at each excitation wavelength (340 and 380 nm). Images were acquired using MetaFluor (Molecular Devices, Ismaning, Germany). Cells were measured in bath solution using 40x magnification (N.A. 1.3) at room temperature. Fura-2 ratios were calculated with ImageJ and the data obtained were analyzed with the Matlab script PeakCaller (36). For all experiments *n* = 6.

### RNA Isolation

For qPCR experiments, human CMs were seeded at a density of 6.3 × 10^4^ cells/cm^2^ on a 24-well plate and were treated with 100 ng/ml Midkine for 6 h at 37°C and 7% CO_2_. Cells were lysed with RLY lysis buffer (Meridian Bioscience, Cincinnati, OH, USA), containing 10 μl/ml β-mercaptoethanol (Sigma Aldrich, St. Louis, MO, USA). RNA isolation from cell lysates was performed by using ISOLATE II RNA Mini Kit (Meridian Bioscience, Cincinnati, OH, USA). Remaining DNA was digested by DNase I (Meridian Bioscience, Cincinnati, OH, USA) for 15 min at RT as recommended by the manufacturer.

### Reverse Transcribed Quantitative Polymerase Chain Reaction (RT-qPCR)

The respective RNA samples were reverse transcribed in cDNA using SuperScript® IV VILO® MasterMix (Life Technologies, Carlsbad, CA, USA). For cDNA transcription, 1–5 ng/ml mRNA were used, and experiment was performed according to manufacturer's instructions. For quantitative PCR, the PowerUp® SYBR® Green Master Mix (Applied Biosystems, Waltham, MA, USA) was used. All procedures were performed according to the manufacturers' instructions. For qPCR, the QuantStudio3 (Applied Biosystems, Waltham, MA, USA) system was utilized. Five-hundred to seven-hundred ng/ml cDNA were used for quantitative PCR. Quantitative mRNA expression of human *troponin I* (for: 5′-CCTCCAACTACCGCGCGCTTAT-3′, rev: 5′-CTGCAATTTTCTCGAGGCGG-3′), *sarco/endoplasmic reticulum Ca*^2+^*-ATPase (SERCA2a)* (for: 5′-CTCCTTGCCCGTGATTCTCA-3′, rev: 5′-CCAGTATTGCAGGTTCCAGGT-3′), *ryanodine receptor 1 (RyR1)* (for: 5′-GGGTTCCTGCCCGACATGAG-3′, rev: 5′-GCACAGGTAGCGGTTCACG-3′), Na^+^/Ca^2+^ exchanger (NCX) (for: 5′-GCCTGGTGGAGATGAGTGAG-3′, rev: 5′-ACAGGTTGGCCAAACAGGTA-3′), *toll-like receptor 4 (TLR4)* (for: 5′-CCTGCGTGGAGGTGTGAAAT-3′, rev: 5′-CTGGATGGGGTTTCCTGTCAA-3′), *toll-like receptor 9 (TLR9)* (for: 5′-AGACCTGAGGGTGGAAGTGT-3′, rev: 5′-CTGGATAGCACCAGTAGCGG-3′) and *purigenic receptor subtype 7 (P2X7)* (for: 5′-CACACCAAGGTGAAGGGGAT-3′, rev: 5′-GGTGTAGTCTGCGGTGTCAA-3′) was examined and calculated by the cycle threshold method ΔΔCt. Respective genes were normalized to expression of the housekeeping gene *glutaraldehyde-phosphate dehydrogenase (GAPDH)* (forward: 5′-TCTCTGCTCCTCCTGTTCGAC-3′, reverse: 5′-CCAATACGACCAAATCCGTTGA-3′) in order to exclude variations. Quantitative mRNA expression was determined by the double-threshold method (ΔΔCT). Results are presented as mean fold change. For all experiments *n* = 6.

### Reactive Oxygen Species (ROS)

For analysis of cellular ROS, human CMs were seeded at a density of 6.3 × 10^4^ cells/cm^2^ on ibidi 12-well slides (ibidi, Germany). Human CMs were treated with 100 ng/ml Midkine for 6 h at 37°C and 7% CO_2_. After treatment, cells were incubated for another 30 min with 5 μM CellROX® Deep Red Reagent (Life Technologies, Carlsbad, CA, USA) at 37°C and 7% CO_2_. Afterwards, cells were fixed with 4% formaldehyde and cell nuclei were stained with Hoechst. Cell were mounted with ProLong® Gold Antifade Mountant. Cells were investigated by blinded investigator by fluorescence microscopy using Axio Imager M.2 microscope and the Zeiss ZEN 2.3 software. Imaging was performed by using 20x magnification (N.A. 0.5). Relative amount of reactive oxygen species was determined by Zeiss ZEN 2.3 software in order to exclude variations. For all experiments *n* = 6.

### Mitochondrial Respiration With Seahorse XF Analyzer

Mitochondrial respiration was analyzed by using the Seahorse XFe96 Analyzer (Agilent Technologies, Santa Clara, CA, USA). This extracellular flux analyzer makes it possible to perform highly accurate real-time measurements of cellular metabolism in living cells by simultaneously quantifying the rates of extracellular acidification (ECAR) and oxygen consumption (OCR), and measuring the glycolysis and the mitochondrial respiration of the cells. For the analysis of mitochondrial respiration, the Seahorse XF Cell Mito Stress Test Kit (Agilent Technologies, Santa Clara, CA, USA) was used. The Seahorse XF Cell Mito Stress Test Kit is an optimized solution for assessing mitochondrial function. During the experiment, the ECAR and the OCR were continuously measured, gaining the parameter for the basal (baseline) respiration of the mitochondria. Afterwards, 2 μM oligomycin, 1 μM carbonyl cyanide 4-(trifluoromethoxy) phenylhydrazone (FCCP), and 0.5 μM antimycin A and rotenone were pneumatically injected into the media of the cells. After automatically and gently mixing, the OCR and the ECAR were measured at multiple times after each injection. After the experiment, cells were fixed with 4% formalin at 4°C overnight. Then, cells were stained with 0.3% Janus-Green solution (Sigma Aldrich, St. Louis, MO, USA), washed and resolved with 0.5 M hydrochloric acid. Optical density was measured at 630 nm and OCR values were normalized to OD 630 nm values to exclude variations. Results were evaluated using Seahorse Wave 2.4 software (Agilent Technologies, Santa Clara, CA, USA), gaining the parameter for spare respiratory capacity of the mitochondria. For the analysis of mitochondrial respiration, cells were seeded with a density of 5 × 10^5^ cells/cm^2^ on Seahorse XFe96 analyzer cell culture plates (Agilent Technologies, Santa Clara, CA, USA) and incubated for 6 h with 100 ng/ml Midkine and the above- mentioned procedure was performed. For all experiments *n* = 6.

### CytoSorb® 300 Experiments

For the therapeutic experiments, the CytoSorb® 300 was used (CytosorbensInc., MonmouthJunction, NJ, USA). Therefore, small columns were prepared. An excess of CytoSorb® 300 at the ratio 2:1 (CytoSorb® 300 to plasma samples) was added on the column as recommended by the manufacturers. Human shock room blood plasma samples were added on the columns and were incubated for 6 or 3 h at RT while continuously shaking. For time-doses experiments, different Midkine concentrations (10,000, 5,000, 2,500, 2,000, 1,500, 1,000, 500, 1,000 pg/ml) diluted in PBS with 1% BSA were added on the columns and were also incubated for 6 and 3 h at RT, while continuously shaking. For all experiments *n* = 6.

### Statistical Analysis

All values were expressed as means ± SEM. Data were analyzed by one-way ANOVA followed by Dunnett's or Tukey's multiple comparison test. For the statistical analysis of two groups, unpaired two-tailed students *t-*test was used. *p* ≤ 0.05 was considered statistically significant. GraphPad Prism 7.0 software was used for statistical analysis (GraphPad Software, Incorporated, San Diego, CA, USA).

## Results

### Midkine Plasma Levels in Multiply Injured Humans and Pigs

**Figure 1:**
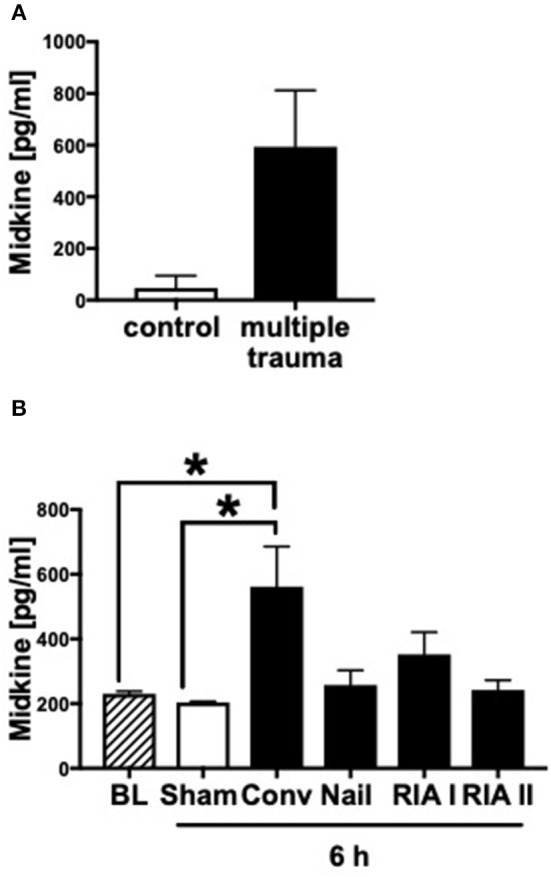
Midkine levels in blood plasma of multiply injured humans and pigs. Midkine levels (pg/ml) in shock room blood plasma from multiply injured patients compared to healthy control group (*n* = 10) **(A)**. Midkine levels (pg/ml) in blood plasma of multiply injured pigs **(B)**. The pigs' femur fracture was either treated with femoral nailing (nailing, *n* = 5), conventional (conv, *n* = 5), or with reamer irrigator aspirator 1 or 2 (RIA I or RIA II, each *n* = 5). Control animals received sham-procedure (*n* = 5). Evaluation of blood plasma at baseline (BL) and 6 h after trauma. Results are presented as mean ± SEM. Data were analyzed by one-way ANOVA followed by Dunnett's or Tukey's multiple comparison test. Results are significant *p < 0.05.

In humans as well as in pigs, the blood plasma concentrations of Midkine increased after multiple trauma compared to the healthy controls (Figures 1A,B). Animals submitted to reamed femoral nailing showed significantly higher Mdk levels when compared with pigs treated with conventional femoral nailing or with reamer irrigator aspirator treatment (RIA I/II; Figure 1B). This indicates that Mdk levels correlate with the invasiveness of the reaming method. In multiply injured pigs, plasma Mdk levels increased significantly after 6 h in the group with conventional reaming of the fracture compared to the control group.

**Figure 2:**
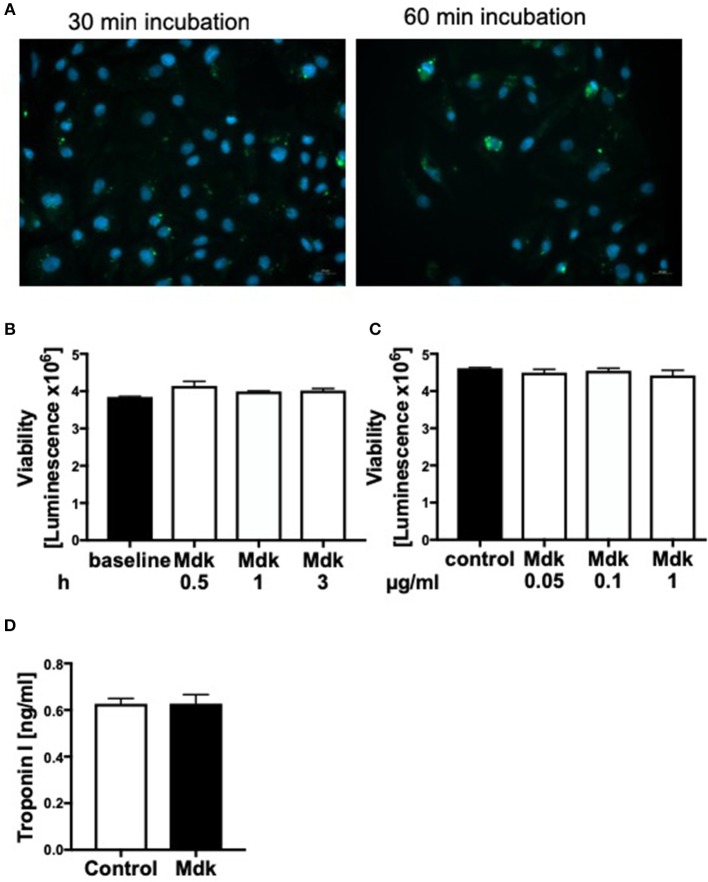
Effects of Midkine on human cardiomyocytes. Immunofluorescence staining of human cardiomyocytes **(A)**. Human cardiomyocytes were treated for 30 and 60 min with 100 ng/ml fluorescein isothiocyanate (FITC)-labeled Midkine (green). Cell nuclei were counterstained with Hoechst (blue). Cell viability of human cardiomyocytes (Luminescence in counts/sec) treated for 0.5, 1, and 3 h with 100 ng/ml Midkine **(B)**. Cell viability of human cardiomyocytes (Luminescence in counts/sec) treated for 3 h with 0.05, 0.1, and 1 μg/ml Midkine **(C)**. Troponin I (ng/ml) in supernatant of human cardiomyocytes, treated for 6 h with 100 ng/ml Midkine **(D)**. Results are presented as mean ± SEM. For all experiments *n* = 6. Data were analyzed by two-tailed, unpaired students *t-*test.

Since plasma Mdk levels increased after multiple trauma, we investigated whether Mdk affects human cardiomyocytes (CMs). After 30 and 60 min the Mdk was actively absorbed into the human CMs and was primarily located around their nucleus *in vitro* (Figure 2A).

### Cell Viability, Cell Damage, and Calcium Handling of Human Cardiomyocytes

**Figure 3:**
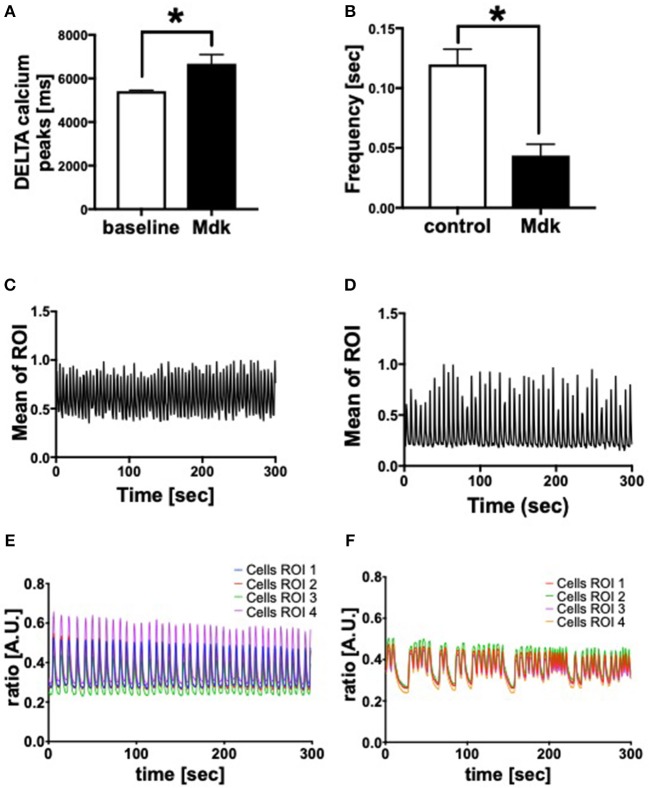
Calcium handling of human cardiomyocytes. Delta calcium peaks (msec) of human cardiomyocytes treated for 30 min with 100 ng/ml Midkine **(A)**. Frequency of calcium signals (sec) of human cardiomyocytes treated for 60 min with 100 ng/ml Midkine **(B)**. Traces of calcium signals of human cardiomyocytes (Mean Calcium peaks of ration of interest (ROI) vs. time in sec) **(C,D)**. Traces of calcium signals of human cardiomyocytes (Ratio of Fura-2 signals in A.U. vs. time in sec) **(E,F)**. Different colors for selected ROI of calcium signals of the cells. For all experiments *n* = 6. Results are presented as mean ± SEM. Data were analyzed by one-way ANOVA followed by Dunnett's or Tukey's multiple comparison test. Results are significant *p < 0.05.

Given that Mdk is actively taken into the cells, we examined whether it then affects the cell viability of the human CMs. The cell viability of the human CMs was neither affected by different Mdk concentrations nor by different incubation times (Figures 2B,C). Furthermore, there were no differences in troponin I concentrations in supernatant of the humans CMs treated with Mdk compared to control cells after 6 h (Figure 2D). However, the calcium handling of the human CMs was altered after Mdk treatment, which is exemplified by the significant increase in their delta calcium peaks (Figure 3A), meaning the cells beat slower in presence of Mdk. Moreover, the frequency of calcium signals in human CMs decreased significantly in presence of Mdk, developing bradycardic conditions (Figure 3B), which is also demonstrated the traces of the calcium signals of the cells (Figures 3C–F).

### Gene Expression of Human Cardiomyocytes

**Figure 4:**
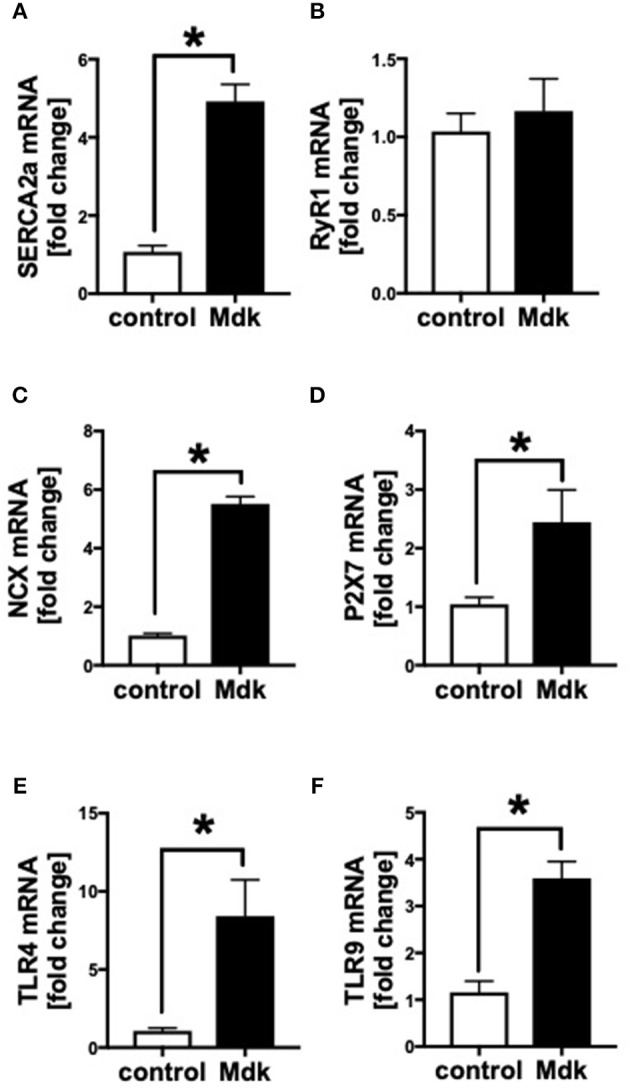
Gene expression of human cardiomyocytes treated for 6 h with 100 ng/ml Midkine. mRNA expression (in fold change) of *sarco/endoplasmatic reticulum Ca*^2+^*-ATPase* (*SERCA2a)* **(A)**, *ryanodine receptor-1 (RyR1)* **(B)**, *sodium-calcium exchanger (NCX)* **(C)**, *purigenic P2X receptor subtype 7 (P2X7)* **(D)**, *toll-like receptor 4 (TLR4)* **(E)**, *toll-like receptor 9 (TLR9)* **(F)**. Results are presented as mean ± SEM. Data were analyzed by two-tailed, unpaired students *t-*test. For all experiments *n* = 6. Results are significant *p < 0.05.

We showed that Mdk alters the calcium handling in human CMs. Next, we investigated the gene expression of specific cardiac calcium pumps as well as the expression of different receptors, which might be involved in Mdk signaling. In human CMs, the mRNA expression of *SERCA2a, NCX, TLR4, TLR9*, and *P2X7* increased significantly in presence of Mdk compared to control (Figures 4A,C–F), indicating for direct effects of Mdk on gene expression of calcium handling proteins. Moreover, the effects of Mdk might be mediated via TLR-P2X7 signaling. The mRNA expression of *RyR1* was unaffected (Figure 4B).

### Mitochondrial Respiration of Human Cardiomyocytes

**Figure 5:**
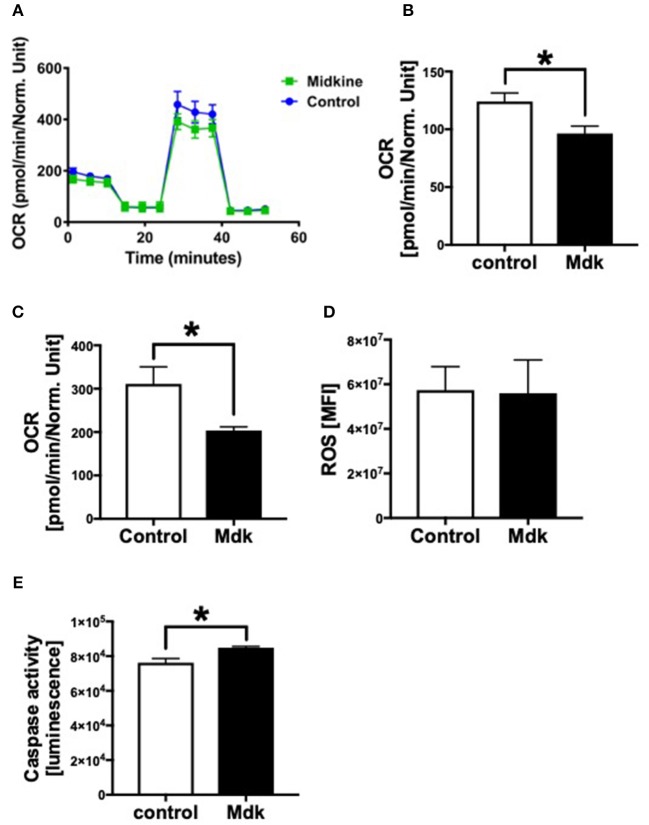
Mitochondrial respiration, cellular reactive oxygen species (ROS) and Caspase 3/7 activity of human cardiomyocytes treated for 6 h with 100 ng/ml Midkine. Oxygen consumption rate (OCR) of human cardiomyocytes (Control, Midkine) during Seahorse MitoStress Assay (OCR in pmol/min/E630 vs. time in min) **(A)**. Basal respiration of human cardiomyocytes (OCR in pmol/min/E630) **(B)**. Spare respiratory capacity of human cardiomyocytes (OCR in pmol/min/E630) **(C)**. Amount of reactive oxygen species (mean fluorescence intensity, MFI) **(D)**. Caspase 3/7 activity (Luminescence in counts/sec) **(E)**. Results are presented as mean ± SEM. For all experiments *n* = 6. Data were analyzed by two-tailed, unpaired students *t-*test. Results are significant *p < 0.05.

In addition, we analyzed the effects of Mdk on the mitochondrial respiration of CMs Figure 5A. The basal respiration as well as the spare respiratory capacity of the human CMs decreased significantly after the Mdk treatment (Figures 5B,C), indicating detrimental effects of Mdk on mitochondrial respiration.

### Intracellular Reactive Oxygen Species (ROS) and Caspase3/7 Activity

As Mdk alters mitochondrial respiration and ATP production of the cells, we next investigated whether Mdk also affects the redox balance of the human CMs. The amount of ROS did not change in human CMs after being treated with Mdk compared to control cells (Figure 5D). Although Caspase3/7 activity increased significantly in human CMs in presence of Mdk (Figure 5E), indicating for enhanced apoptosis in the cells.

### Filtration of Midkine by CytoSorb® 300

**Figure 6:**
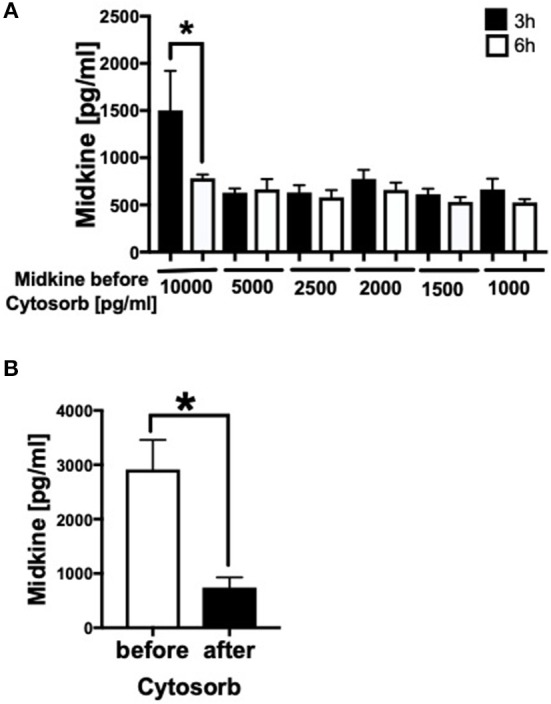
Filtration of Midkine by CytoSorb® 300. Filtration of different Midkine concentrations (10,000, 5,000, 2,500, 2,000, 1,500, 1,000 pg/ml) (*n* = 6) with CytoSorb® 300 after 3 h (black bars) and 6 h (white bars) **(A)**. Midkine concentration before and after filtration with CytoSorb® 300 on the x-axis in pg/ml. Midkine levels (pg/ml) in human shock room blood plasma of multiply injured patients before and after filtration with CytoSorb® 300 (*n* = 11) **(B)**. Results are presented as mean ± SEM. Data were analyzed by one-way ANOVA followed by Dunnett's or Tukey's multiple comparison test. Results are significant *p < 0.05.

Because Mdk is elevated in plasma of multiply injured humans and pigs and acts on human CMs, we examined the potential of a therapeutic approach: the absorption capacity of Mdk from human blood by CytoSorb® 300. After incubation of different Mdk concentrations with CytoSorb® 300, the Mdk levels decreased between 45 and 95% within 6 h (Figure 6A). Especially high Mdk concentrations (10,000 pg/ml) were significantly reduced up to 95% after filtration with CytoSorb® 300 after 6 h compared to the 3 h incubation (Figure 6A). Moreover, Mdk levels in plasma from multiply injured patients were significantly reduced after incubation with CytoSorb® 300 (Figure 6B).

## Discussion

Our study shows for the first time that Mdk is elevated in blood circulation after multiple trauma. This elevation is similar to other traumatic injuries, suggesting that circulating Mdk may act as a novel inflammatory marker for polytrauma (8, 9, 11). Furthermore, we demonstrated that Mdk acts directly on human cardiomyocytes *in vitro* and is actively taken up by these cells, altering their functionality without affecting their viability. We found that Mdk affects the functionality of the human CMs by altering their calcium handling. The delta calcium peaks of the human CMs increased significantly after Mdk treatment, meaning the cells became bradycardic. Moreover, the frequency of the calcium signals in human CMs decreased significantly after Mdk treatment, confirming the bradycardic effect of Mdk. The mRNA expression of the specific cardiac calcium pumps *SERCA2a* and *NCX* also increased significantly after Mdk treatment, suggesting direct effects of Mdk on calcium handling in the cells. The location of Mdk around the cell nucleus of the human CMs confirmed the regulatory effects on cellular gene expression of the calcium handling proteins. Alterations in calcium signals as well as in mRNA expression of *SERCA2a* and *NCX* were also described previously in presence of other trauma-related inflammatory biomarkers and DAMPs as well as in different trauma models and during sepsis, nominating Mdk as a powerful cardio-depressive mediator after trauma and during sepsis (25, 37–42). However, cardiac overexpression of SERCA2a in rodents improved cardiac contractility and relaxation, which might also indicate potential protective effects of Mdk in the heart, which would require to be investigated in future studies (43, 44). We also novelly showed that the basal respiration as well as the spare respiratory capacity of the mitochondria of the human CMs decreased significantly, indicating detrimental effects of Mdk on cellular mitochondrial respiration and energy production. Nevertheless, the amount of cytosolic reactive oxygen species (ROS) was not altered in the human CMs in presence of Mdk. Mitochondrial dysfunction was also depicted previously for other trauma-related biomarkers (7, 45, 46). The detrimental and cardio-depressive effects of Mdk on the human CMs might be mediated *via* the toll-like receptor (TLR) 4, TLR9, and the pyrogenic receptor subtype 7 (P2X7) since the mRNA expression of these receptors was significantly upregulated. All of these receptors have been demonstrated to be involved in the DAMP-associated cardiac signaling pathways in different trauma models (47, 48). The activation of the TLRs results in increased cardiac inflammation, mediated via the nuclear factor κ B (NFκB) (47). This TLR-mediated cardiac inflammation leads to cardiac injury and finally results in cardiac contractile dysfunction (47–49). Since the mRNA expression of the TLRs was upregulated, the protein expression of these receptors might be increased after Mdk treatment. This might lead to sensitization of the CMs for other systemic circulating DAMPs, such as HMGB1 and extracellular histones, which were elevated after polytrauma, leading to cardiomyocyte dysfunction (7, 24, 50). The P2X7 receptor was also shown to be involved in cardiac contractile dysfunction (51). Interestingly, the Caspase 3/7 activity increased in human CMs after treatment with Mdk, which was demonstrated previously in cardiac tissue *in vivo* in an experimental polytrauma model in pigs (41). So far, Mdk was described as an anti-apoptotic factor by decreasing caspase activity in other cells, such as neurons and HepG2 cells, which is in accordance to unaffected cell viability in the present study (52, 53). The effects of Mdk on cellular apoptosis of human CMs has not been described, so far. In contrast to other cells, human CMs seem to follow different cellular processes and various signal cascades might be involved in Caspase-3/7 activation and activity when these cells were treated with Mdk. Moreover, this phenomenon could also be time-dependent as we solely investigated the Caspase-3/7 activity after 6 h of Mdk exposure. This observation should be the subject of future studies in order to understand the specific effects of Mdk on apoptosis of human CMs.

Therapeutic approaches treating post-traumatic cardiac dysfunction are still limited. In this study, we clearly showed that Mdk is elevated in plasma after multiple trauma and is predominantly detrimental on human CMs, causing the development of post-traumatic cardiac dysfunction. As a consequence, we investigated the efficiency of CytoSorb® 300 in filtering Mdk from human blood plasma. CytoSorb® 300 is an absorption column, composed of porous polymer beads, which is normally used in the intensive care unit (ICU) for septic patients or for patients with SIRS. The filtration potential of CytoSorb® 300 for various trauma-associated cytokines and DAMPs was already demonstrated by others (31, 54). Here, we showed for the first time that CytoSorb® 300 is able to absorb Mdk dose-dependently, filtering high Mdk concentrations (10,000 pg/ml) up to 95%. Moreover, CytoSorb® 300 filtered Mdk from human plasma obtained on admission to the emergency room, making it a very promising therapeutic approach for treatment and prevention of post-traumatic cardiac dysfunction. One huge benefit of using CytoSorb® 300 instead of single antibodies for therapy is that CytoSorb® 300 is able to filter a high amount of many miscellaneous damage- and inflammation molecules after trauma and not only a single molecule, which is the case of antibody treatment. Furthermore, filtration of Mdk by CytoSorb® 300 might limit other negative effects of Mdk on polytrauma patients, since it was shown that Mdk acts as an inhibitor of fracture healing and that high Mdk serum levels were associated with poor outcome in septic patients. Finally, we found that systemic Mdk is higher after conventional reaming, compared to nailing without reaming and to RIA I/II. Consequently, treatment of the fracture with RIA I/II might be better for fracture outcome as well as for fracture healing after trauma (55). In addition, conventional reaming of the fracture might have other negative effects after trauma (e.g., pulmonary embolism).

One limitation of the study might be the small sample size (*n* = 6) to investigate different treatment approaches for the femur fracture. Consequently, more experiments are needed to find the best and the least invasive treatment approach. The same applies for a possible correlation between fracture treatment approaches and systemic Mdk levels. Because investigated groups were heterogenous, a bigger number of samples might be helpful to extrapolate the results to a clinical population. Another limitation might be that we only used small columns with Cytosorb® 300 polymer beads in our study, trying to mimic the clinical application in ICU. However, as our study was only an experimental approach, clinical studies should be performed, including more patients and larger application approaches of Cytosorb® 300. This may help to- confirm the therapeutic potential of Cytosorb® 300 for the prevention of post-traumatic cardiac dysfunction by filtering Mdk from human blood *in vivo*. Furthermore, it is not possible to mimic *in vitro* the real *in vivo* inflammatory conditions, which occur after trauma. The presence of many different inflammatory mediators and DAMPs and the activation of different signal cascades in the cells lead to post-traumatic cardiac dysfunction. Consequently, it is not possible to specify these detrimental effects on one single mediator like Mdk.

**Figure 7:**
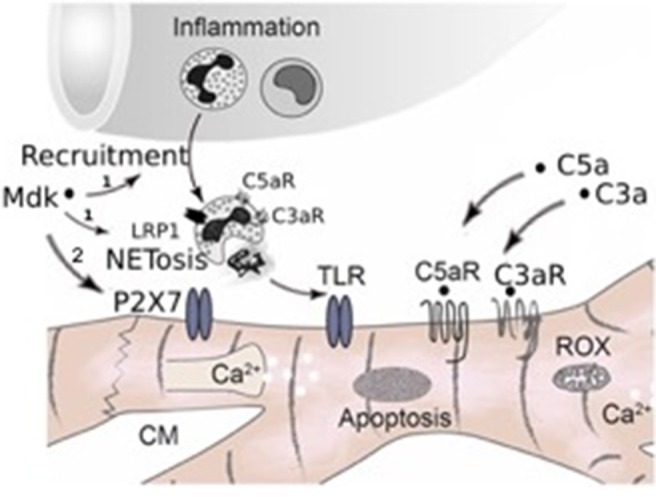
Schematic representation of systemic inflammation after trauma and during sepsis. After trauma Midkine is released systemically. Midkine recruits polymorphonuclear neutrophils (PMNs) and induces the release of extracellular traps (NETosis) via the receptor-related protein 1 (LRP1) on the neutrophils, which was demonstrated by Weckbach et al. (23), (1). The neutrophil extracellular traps (NETs) include extracellular histones, which act via toll-like receptors (TLRs) on the surface of cardiomyocytes, inducing cardiac dysfunction and cardiac damage. Furthermore, Midkine is able to act detrimental on human cardiomyocytes by direct interactions via TLR4, TLR9, and pyrogenic receptor subtype 7 (P2X7), inducing enhanced apoptosis, disturbing calcium signaling and impairing mitochondrial respiration (2, Hypothesis of this manuscript). Additionally, after trauma and during sepsis the complement factors C5a and C3a are released systemically. Both act directly via their receptors (C5aR, C3aR) on cardiomyocytes, leading to cardiac dysfunction. Moreover, the complement factors also induce NETosis of neutrophils via their receptors.

Taken together, in our study we observed for the first time that Mdk is elevated systemically after multiple trauma in humans and pigs, acting cardio-depressive on human CMs by impairing their calcium handling and mitochondrial respiration capacity *in vitro*. PlX27/TLR might be involved in mediating these detrimental effects of MdK (Figure 7). In the clinical setting, the hemadsorption filter Cytosorb® 300 might be a powerful tool to remove cardio-depressive mediators from patients' circulation and therefore help to improve cardiac function.

## Data Availability

All datasets generated for this study are included in the manuscript and/or the supplementary files.

## Ethics Statement

Human plasma from 11 multiply injured patients with a history of acute blunt or penetrating trauma and an ISS ≥ 16 was collected after hospital admission in the University Hospital of the Goethe-University Frankfurt with institutional ethics committee approval (312/10), in accordance with the Declaration of Helsinki and following the Strengthening the Reporting of Observational studies in Epidemiology (STROBE)-guidelines (32). All enrolled patients signed the written informed consent form themselves or written informed consent was obtained from the nominated legally authorized representative on the behalf of participants in accordance with ethical standards.

The animal housing and experimental protocols were approved by the Cantonal Veterinary Department, Zurich, Switzerland, under license no. ZH 138/2017, and were in accordance with Swiss Animal Protection Law. Housing and experimental procedures also conformed to the European Directive 2010/63/EU of the European Parliament and of the Council on the Protection of vertebrate animals used for scientific purposes (Council of Europe no. 123, Strasbourg 1985) and to the Guide for the Care and Use of Laboratory Animals (Institute of Laboratory Animal Resources, National Research Council, National Academy of Sciences, 2011).

## Author Contributions

IL, BW, MB, TE, GF, SH, ML, and NC performed the experiments including animal studies, cell culture experiments, microscopic studies, and ELISAs. IL primarily wrote the paper. MH-L, FG, BR, IM, RP, H-CP, and MK contributed to experimental design and data analysis and coordinated the study and supervised financial support for the studies. All authors made substantial contributions to conception and design of the study, participated in drafting the article, and gave final approval of the version to be published.

## TREAT Research Group

Auner B, Stormann P, Simon TP, Marx G, Haug A, Egerer L, Giensven MV, Huber-Lang M, Tolba R, Reiss K, Uhlig S, Horst K, Teuben M, Almahoud K, Kalbas Y, Luken H, Almahoud K, and Hildebrand F.

### Conflict of Interest Statement

The authors declare that the research was conducted in the absence of any commercial or financial relationships that could be construed as a potential conflict of interest.
